# Vascular Complications in Chronic Active Epstein–Barr Virus Disease: Clinical Spectrum, Pathogenic Mechanisms, and Potential Disease Modifiers

**DOI:** 10.3390/ijms27219404

**Published:** 2026-10-22

**Authors:** Jong-Woan Kim, Woo Sub Yang, Minji Park, Hyun Jung Koh

**Affiliations:** 1 Department of Anesthesiology and Pain Medicine, Seoul St. Mary’s Hospital, College of Medicine, The Catholic University of Korea, Seoul 06591, Republic of Korea; jwjw12@naver.com (J.-W.K.); parkmj9315@gmail.com (M.P.); 2 Department of Genetics, Yale Stem Cell Center, Yale School of Medicine, New Haven, CT 06520, USA; woosubyang@gmail.com

**Keywords:** acute kidney injury, chronic active Epstein–Barr virus disease, endothelial dysfunction, hyperinflammation, intracranial hemorrhage, vascular complications

## Abstract

Chronic active Epstein–Barr virus disease (CAEBV) is a rare, potentially life-threatening EBV-positive T/NK-cell lymphoproliferative disorder characterized by persistent systemic inflammation and multiorgan involvement. Although hemophagocytic lymphohistiocytosis and progression to lymphoid malignancy are well-recognized complications, vascular manifestations—including vasculitis, arterial dilatation and aneurysm formation, intracranial hemorrhage, and vascular involvement of multiple organ systems—remain poorly characterized. Available evidence suggests that persistent activation of EBV-infected T/NK cells, cytokine-mediated hyperinflammation, endothelial injury, and coagulation abnormalities may contribute to CAEBV-associated vasculopathy, whereas the roles of complement activation and thrombotic microangiopathy remain less well established. This review synthesizes the clinical spectrum and proposed mechanisms of vascular complications in CAEBV, with particular attention to the strength of evidence supporting individual pathogenic pathways. Improved recognition of vascular manifestations, application of contemporary CAEBV diagnostic criteria, and further mechanistic and multicenter studies are needed to improve risk stratification and management.

## 1. Introduction

### 1.1. Chronic Characteristics of Adult-Onset CAEBV

Epstein–Barr virus (EBV), a ubiquitous human herpesvirus, infects more than 90% of the global adult population and establishes lifelong latent infection following primary exposure, which is usually asymptomatic or presents as self-limited infectious mononucleosis (IM). In a small subset of individuals, impaired immune control permits persistent proliferation of EBV-infected T or NK cells, resulting in chronic active EBV disease (CAEBV), a rare but life-threatening EBV-positive T/NK-cell lymphoproliferative disorder [1,2]. Updated diagnostic criteria define CAEBV as a systemic disease characterized by persistent IM-like symptoms, elevated EBV DNA levels (≥10,000 IU/mL in whole blood) and evidence of EBV-infected T or NK cells, rather than as a chronic viral infection alone [3].

Although CAEBV has traditionally been regarded as a pediatric disease, adult-onset CAEBV is increasingly recognized as a distinct clinical presentation that may be associated with a more aggressive clinical course and poorer prognosis than pediatric-onset disease. Compared with pediatric patients, adults more frequently present with atypical clinical manifestations, including less prominent IM-like symptoms but greater extranodal involvement affecting the pulmonary, cardiovascular, cutaneous, renal, and central nervous system. T- or NK-cell CAEBV occurs predominantly in East Asia and Latin America [1,4] and is associated with substantially higher mortality, mainly because of progression to hemophagocytic lymphohistiocytosis (HLH), liver failure, lymphoma, and multiorgan dysfunction [5].

### 1.2. Contemporary Definition of CAEBV and Distinction from Other EBV-Associated Disorders

Chronic active Epstein–Barr virus infection (CAEBV) is not synonymous with EBV reactivation or isolated elevation in EBV DNA levels. In the fifth edition of the World Health Organization Classification of Hematolymphoid Tumors, CAEBV is classified among EBV-positive T- and NK-cell lymphoid proliferations and lymphomas and termed “systemic chronic active EBV disease” [6]. According to contemporary diagnostic criteria, CAEBV is characterized by persistent or recurrent infectious mononucleosis-like symptoms, an increased EBV DNA load, and the presence of EBV-infected T or NK cells in peripheral blood and/or affected tissues [3]. EBV-associated hemophagocytic lymphohistiocytosis (EBV-HLH), by contrast, represents a hyperinflammatory syndrome that may occur in the setting of primary EBV infection, reactivation, or underlying CAEBV, but does not by itself fulfill the criteria for CAEBV.

In this review, reports were interpreted according to the level of diagnostic certainty. Studies fulfilling contemporary diagnostic criteria were considered CAEBV-specific evidence, whereas older reports lacking sufficient information to verify current criteria were treated as historical or incompletely characterized cases. Vascular manifestations associated with acute EBV infection, EBV reactivation, or other EBV-associated disorders were considered indirect evidence and were not regarded as CAEBV-specific vascular manifestations.

### 1.3. Vascular Complications of CAEBV: Current Knowledge Gaps and Scope of This Review

Although HLH has been extensively reviewed, vascular complications—including vasculitis, aneurysm formation, and intracranial hemorrhage—remain poorly characterized and have been described primarily in isolated case reports and small case series [7,8]. Renal dysfunction, including acute kidney injury, may coexist with these complications but does not necessarily indicate vascular injury. Consequently, their underlying biological mechanisms have not been comprehensively synthesized, limiting current understanding of the pathogenesis of severe vascular injury in CAEBV. The rarity and clinical heterogeneity of CAEBV further limit the availability of high-quality evidence, making early recognition and appropriate management challenging [3].

Available evidence suggests that persistent immune activation, endothelial dysfunction, thrombo-inflammatory pathways, and coagulation abnormalities may contribute to vascular injury in CAEBV, while the potential contribution of complement activation remains less well established. However, the relative contribution and interactions of these mechanisms remain poorly defined.

The aim of this review is to critically synthesize current experimental and clinical evidence regarding the molecular mechanisms, clinical manifestations, diagnostic challenges, and therapeutic considerations of vascular complications associated with CAEBV. Particular attention is given to immune-mediated endothelial injury, complement and coagulation abnormalities, and thrombo-inflammatory pathways.

### 1.4. Literature Search and Evidence Assessment

This narrative review was informed by a focused literature search conducted using PubMed/MEDLINE, Scopus, and Google Scholar for studies published until August 2026 that were relevant to CAEBV, its vascular manifestations and pathogenic mechanisms. Search terms included combinations of (“chronic active Epstein–Barr virus” OR “CAEBV”) AND (“vascular” OR “vasculitis” OR “aneurysm” OR “endothelial” OR “vascular injury” OR “microangiopathy” OR “complement”), with additional searches combining (“chronic active Epstein–Barr virus” OR “CAEBV” OR “Epstein–Barr virus” OR “EBV”) with (“postpartum” OR “pregnancy”). Relevant original studies, case series, case reports, reviews, and consensus or guideline articles were considered. Additional publications were identified from the reference lists of relevant articles.

Because CAEBV diagnostic criteria have evolved over time, reports were interpreted with consideration of contemporary criteria, particularly the demonstration of EBV-infected T or NK cells when reported. Older reports in which contemporary criteria could not be verified were retained only when clinically informative and were interpreted as historical or incompletely characterized evidence rather than definitive CAEBV-specific evidence. Reports of vascular disease associated with acute EBV infection or other EBV-associated conditions were used only as indirect mechanistic or clinical context. Given the limited disease-specific evidence, postpartum-related literature was considered primarily as biological and clinical context rather than as direct evidence of CAEBV pathogenesis.

## 2. Clinical Spectrum of Vascular Complications in CAEBV

### 2.1. Overview of Clinical Manifestations

Vascular complications represent uncommon but potentially life-threatening manifestations of chronic active Epstein–Barr virus (CAEBV) infection [8,9]. Most reported patients have been children or young adults. Vascular involvement may affect multiple territories, including the coronary and systemic arteries [7,8,10], cerebral circulation [8,11], pulmonary vasculature [12,13,14], and cutaneous vessels [14,15]. These complications may occur in the setting of severe systemic inflammation and can accompany hemophagocytic lymphohistiocytosis (HLH) and multi-organ dysfunction in advanced disease [16].

Reported vascular manifestations encompass inflammatory vasculitis or vasculopathy, coronary and systemic arterial aneurysms, pulmonary vascular involvement, and cerebral aneurysmal disease with potentially fatal hemorrhagic complications [8,12,17]. Their heterogeneous clinical presentation may complicate early recognition, particularly in adult-onset disease [16]. Table 1 summarizes the reported vascular manifestations of CAEBV across major organ systems.

### 2.2. Cardiovascular Manifestations

#### 2.2.1. Coronary Artery Abnormalities

Coronary artery abnormalities are among the best-characterized vascular complications of CAEBV. Reported lesions include coronary artery dilatation, single or multiple coronary artery aneurysms, giant coronary aneurysms, and in some cases, associated thrombosis [7,10]. Although coronary artery lesions have been reported more frequently in pediatric patients, similar abnormalities have also been described in adult CAEBV [7].

#### 2.2.2. Large-Vessel Involvement

In addition to coronary arteries, aneurysmal or inflammatory involvement of the aorta, sinus of Valsalva, and other systemic arteries has been reported [7,10]. In some patients, multiple arterial territories are affected, supporting that vascular involvement may occur as part of a broader systemic vasculopathy rather than as an isolated vascular lesion [7].

### 2.3. Renal Involvement and Its Relationship with Vascular Injury

Renal manifestations have been reported in CAEBV but are heterogeneous and should not be regarded as evidence of vascular injury per se. Reported renal findings include renal dysfunction, proteinuria, hematuria, and histopathologically documented renal involvement, including tubulointerstitial lesions and IgA nephropathy [22,23].

### 2.4. Cerebrovascular Manifestations

Cerebrovascular involvement is an uncommon but potentially severe manifestation of CAEBV and has been associated with poor outcomes [20]. Reported cerebrovascular manifestations include cerebral vasculitis or vasculopathy, intracranial aneurysm formation, and intracranial hemorrhage, including fatal hemorrhage following aneurysmal rupture [8,11,21]. In some patients, cerebrovascular lesions occur in the context of broader systemic vascular involvement. For example, multisystemic aneurysmal disease involving the coronary, abdominal, cerebral, and vertebral arteries has been reported in CAEBV, with fatal cerebral aneurysm rupture [8]. These observations suggest that cerebrovascular complications may occur as part of systemic vasculopathy rather than as isolated CNS disease, although the underlying mechanisms remain incompletely understood.

### 2.5. Cutaneous Vascular Manifestations

Cutaneous vascular involvement has occasionally been reported in systemic CAEBV, including vasculitic lesions, recurrent ulcers, and subcutaneous nodules. Histopathological examination of affected skin may demonstrate infiltration by EBER-positive T/NK cells and, in selected cases, vasculitic vascular injury. However, evidence specifically linking vasculitic ulcers or subcutaneous nodules to systemic CAEBV remains limited and is derived largely from individual case reports [14,15].

### 2.6. Postpartum Immune Adaptation as a Potential Clinical Modifier

The postpartum period is characterized by gradual restoration of pregnancy-associated immune adaptations, including changes in cell-mediated immunity and inflammatory responses [24]. Changes in EBV-specific immune control have also been observed during pregnancy and postpartum [25], providing a biological rationale for considering this period as a potential modifier of pre-existing EBV-associated immune dysregulation. However, direct evidence that postpartum immune changes promote expansion of EBV-infected T/NK cells, trigger CAEBV progression, or contribute to CAEBV-associated vascular injury is currently lacking. Available reports of CAEBV-associated HLH during pregnancy, postpartum EBV-HLH, and pregnancy in patients with previously treated CAEBV [26,27,28] do not establish a causal relationship between postpartum immune adaptation and CAEBV exacerbation. Therefore, postpartum immune adaptation should be regarded only as a potential clinical modifier rather than an established pathogenic trigger. When CAEBV is suspected in the postpartum setting, diagnosis should follow contemporary criteria, including demonstration of EBV-infected T or NK cells, rather than relying on postpartum status, elevated EBV DNA, or nonspecific inflammatory manifestations alone [3].

## 3. Molecular Mechanisms Underlying Vascular Injury in CAEBV

### 3.1. Viral Latency Programs and Aberrant Intracellular Signaling

CAEBV is characterized by the persistent expansion and survival of EBV-infected T/NK cells, accompanied by dysregulated viral and host signaling [29,30]. EBV latent gene products can activate intracellular pathways involved in cell survival, proliferation, and inflammatory responses. In particular, latent membrane protein 1 (LMP1) can activate NF-κB, JAK/STAT, PI3K/AKT, and MAPK signaling through TRAF- and TRADD-dependent mechanisms, thereby promoting pro-survival and pro-inflammatory responses [30,31,32,33,34]. Activation of these pathways may enhance the production of inflammatory cytokines, including IL-1β, IL-6, TNF-α, and IFN-γ, and contribute to sustained immune activation [30,32,33].

EBV-derived non-coding RNAs, including BamHI-A rightward transcript (BART) microRNAs, have been implicated in the modulation of host immune responses and cellular survival pathways, potentially contributing to the persistence of EBV-infected cells [35,36,37]. Although the specific contribution of individual viral products to vascular injury in CAEBV has not been directly established, persistent activation and survival of EBV-infected T/NK cells may provide a molecular basis for the chronic inflammatory milieu in which endothelial and vascular injury develops [29,30,31,32,33].

### 3.2. Interaction Between EBV-Positive T/NK Cells and Vascular Endothelium

Vascular injury in CAEBV may involve both direct infiltration of EBV-infected lymphocytes into vascular tissues and indirect injury mediated by inflammatory or cytotoxic factors. Histopathologic studies of CAEBV-associated vascular lesions have demonstrated EBV-positive T-lymphocyte infiltration within affected vascular walls. In a fatal case with giant coronary and aortic aneurysms, lymphoid vasculitis involving the coronary arteries, coronary venules, and aorta was accompanied by EBER-positive T-cell infiltration [38]. Similarly, large-vessel arteritis associated with CAEBV has been characterized by T-lymphocyte infiltration around the vasa vasorum, destruction of the medial elastic laminae, severe intimal thickening, and detection of EBV within diseased vascular tissue and infiltrating lymphocytes [39]. Experimental evidence also supports interactions between EBV-positive lymphoid cells and vascular endothelium. EBV-positive NK-cell lines have been shown to adhere to cultured endothelial cells, with adhesion enhanced by inflammatory cytokine stimulation [40]. These findings provide direct histopathologic evidence linking EBV-infected lymphocytes to vascular lesions in at least a subset of patients with CAEBV [38,39].

Activated EBV-infected T/NK cells may also contribute to tissue injury through cytotoxic effector pathways, including Fas/Fas ligand and perforin/granzyme signaling, together with the release of inflammatory cytokines such as IL-1β, IFN-γ, TNF-α, and IL-6 [32,33]. However, the specific contribution of individual cytotoxic pathways to CAEBV-associated vascular injury has not been directly established. These inflammatory mediators can promote endothelial activation and may shift the endothelial surface toward a pro-inflammatory and procoagulant phenotype. In particular, experimental evidence in systemic CAEBV has shown that IL-1β can inhibit endothelial-cell proliferation and increase surface coagulation activity, supporting a potential link between inflammation, endothelial dysfunction, and thrombo-inflammatory vascular injury [41].

Persistent vascular infiltration and inflammation may also contribute to progressive structural damage of the arterial wall. Histopathologic observations of medial injury, elastic lamina destruction, and intimal thickening in CAEBV-associated arteritis provide a potential mechanistic link between chronic vascular inflammation and the arterial dilatation, aneurysm formation, and large-vessel vasculopathy reported in CAEBV [38,39].

### 3.3. Cytokine Network and Inflammatory Vascular Remodeling

Chronic systemic inflammation is a prominent feature of CAEBV and may contribute to vascular injury. Persistent activation of EBV-infected T/NK cells and surrounding immune cells can generate a cytokine-rich inflammatory environment, which may become particularly pronounced in patients who develop hemophagocytic lymphohistiocytosis (HLH) [42,43,44]. Cytokine-driven activation of monocytes may promote tissue factor expression and procoagulant activity in EBV-associated T/NK-cell disorders complicated by HLH [44]. In CAEBV, elevated IL-1β levels have been associated with angiopathy, providing more direct, although still limited, evidence linking inflammatory cytokines to vascular complications [41].

Among the inflammatory mediators implicated in CAEBV-associated vascular injury, IL-1β has the most direct disease-specific evidence. In a study of 17 patients with systemic CAEBV, elevated plasma IL-1β was detected in four patients, three of whom had clinically apparent angiopathy, whereas angiopathy was not observed among patients without detectable plasma IL-1β [41]. In an endothelial-cell model, IL-1β inhibited endothelial proliferation and increased surface coagulation activity, suggesting a potential mechanism through which systemic inflammation may promote endothelial dysfunction and procoagulant changes in CAEBV [41]. However, these findings were derived from a small cohort and require confirmation in larger studies.

Other inflammatory mediators, including IFN-γ, TNF-α, and IL-6, may further contribute to endothelial activation and vascular remodeling [32,33]. Persistent vascular inflammation may in turn promote structural remodeling and compromise vascular wall integrity. Consistent with this possibility, histopathologic studies of CAEBV-associated arteritis have demonstrated destruction of the medial elastic laminae and severe intimal thickening [39]. Together, these inflammatory and structural changes may contribute to arterial dilatation, aneurysm formation, and other vascular abnormalities observed in severe CAEBV [38,39].

In patients with concurrent HLH, cytokine-driven hyperinflammation may aggravate systemic inflammation, coagulopathy, and multiorgan dysfunction [44,45,46]. However, the independent contribution of HLH-associated hyperinflammation to vascular injury in CAEBV has not been established. Current evidence does not clearly distinguish vascular injury directly related to CAEBV from injury secondary to HLH, disseminated intravascular coagulation, or thrombotic microangiopathy [41,44,47].

### 3.4. Complement Activation, Coagulopathy, and Thrombotic Microangiopathy

Direct CAEBV-specific evidence for complement-mediated vascular injury or thrombotic microangiopathy remains limited. In thrombotic microangiopathy (TMA) more broadly, dysregulated complement activation on endothelial surfaces promotes endothelial injury, platelet activation, and microvascular thrombosis, in part through formation of the membrane attack complex (C5b-9). This mechanism is well established in complement-mediated TMA/aHUS and has also been implicated in other secondary TMA syndromes, including pregnancy-associated TMA and HELLP syndrome [48,49,50,51]. Accordingly, the potential contribution of complement activation to CAEBV-associated vascular injury should be regarded as extrapolated rather than established.

Renal involvement in CAEBV appears to be heterogeneous and should not be attributed solely to microvascular injury [22,52]. Case reports have described diverse glomerular lesions, including immune complex-mediated and mesangial proliferative glomerulonephritis, in association with chronic active EBV infection [53,54], whereas other studies have demonstrated tubulointerstitial infiltration by EBV-infected T cells [22,52]. Thus, acute kidney injury or other renal abnormalities in patients with CAEBV do not by themselves establish the presence of TMA or complement-mediated vascular injury. When clinical and/or pathological findings support a TMA phenotype, complement activation may represent one potential amplification pathway; however, this possibility remains largely extrapolated from the broader TMA literature and has not been established as a CAEBV-specific mechanism [48,51].

### 3.5. Integrated Model of CAEBV-Associated Vasculopathy

Taken together, available evidence suggests that vascular involvement in CAEBV may result from several interacting processes rather than a single pathogenic pathway. CAEBV-specific studies support persistent activation of EBV-infected T/NK cells, cytokine-mediated inflammation, and interactions between EBV-infected lymphocytes and vascular endothelial cells [40,41,42]. These processes may promote endothelial activation and inflammatory vascular injury and may contribute to vasculitis, arterial dilatation or aneurysm formation, and severe vascular complications reported in patients with CAEBV [7,8,11,38,39].

However, the strength of evidence differs substantially among the proposed mechanisms. In particular, complement activation and thrombotic microangiopathy are less well established as CAEBV-specific mechanisms and are supported largely by indirect evidence and extrapolation from the broader literature on inflammatory and complement-mediated TMA [48,51].

**Figure 1:**
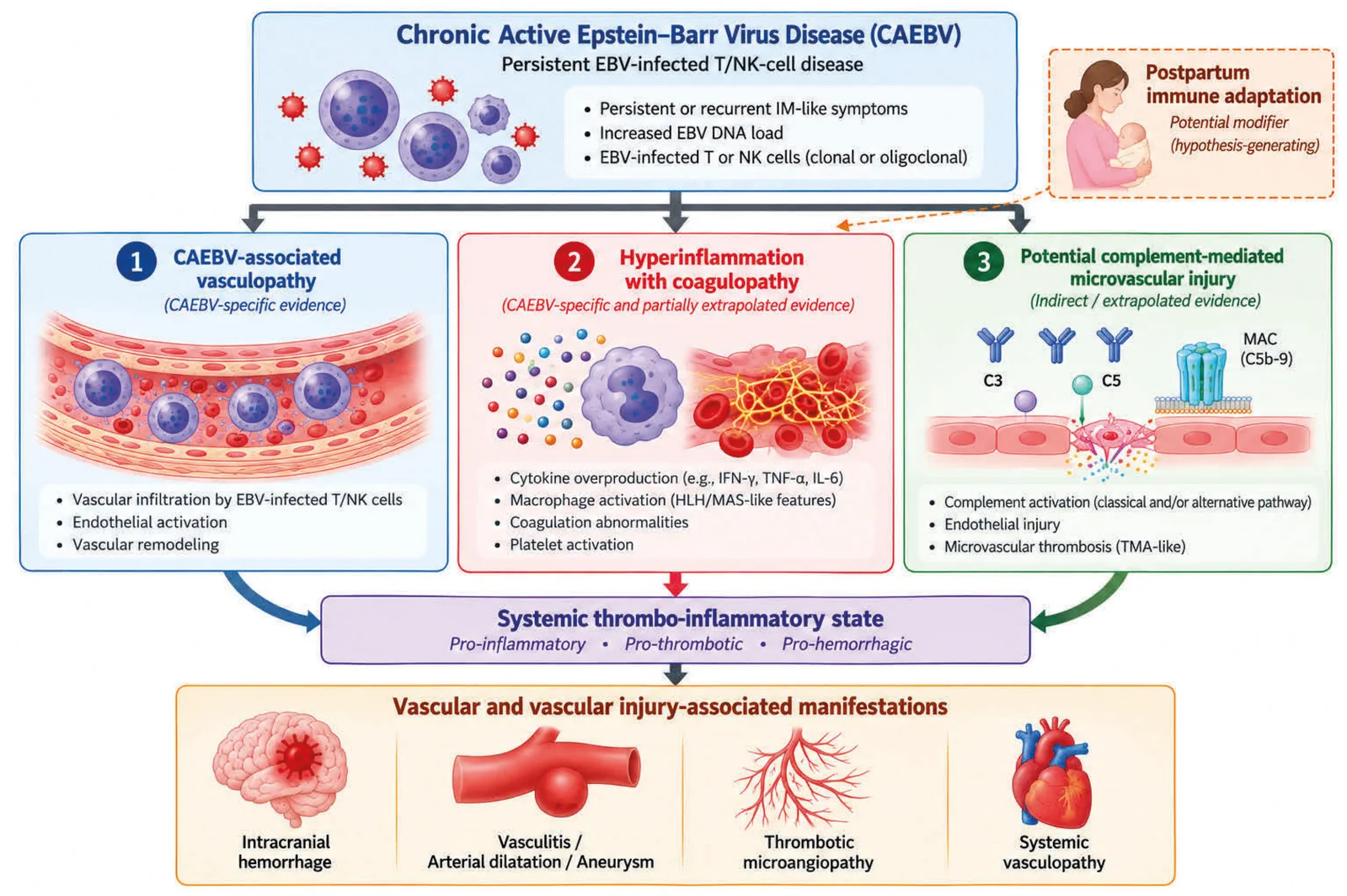
Evidence-graded integrated framework of vascular injury in chronic active Epstein–Barr virus disease (CAEBV). Persistent EBV-infected T or NK cells may contribute to vascular injury through interacting pathways involving CAEBV-associated vasculopathy and hyperinflammation with coagulopathy. Complement-mediated microvascular injury is shown as a potential pathway because direct CAEBV-specific evidence remains limited and is derived largely from extrapolation from other thrombotic microangiopathic and inflammatory conditions. These pathways may converge on a systemic thrombo-inflammatory state and contribute to vascular manifestations, including vasculitis, arterial dilatation or aneurysm formation, thrombotic microangiopathy-like manifestations, and intracranial hemorrhage. Postpartum immune adaptation is shown as a hypothesis-generating potential modifier of the hyperinflammatory pathway rather than an established pathogenic mechanism. Solid arrows indicate the main proposed mechanistic relationships, whereas the dashed arrow denotes the hypothesis-generating potential influence of postpartum immune adaptation.

Collectively, these findings suggest an integrated model in which CAEBV-associated hyperinflammation may contribute to vascular injury, providing a basis for the proposed integrated model [41,42,43]. Figure 1 summarizes these relationships while distinguishing CAEBV-specific evidence from proposed or indirectly supported mechanisms.

## 4. Discussion

Despite increasing recognition of chronic active Epstein–Barr virus (CAEBV) as a systemic EBV-positive T/NK-cell lymphoproliferative disorder, the mechanisms underlying its vascular complications remain incompletely understood. The available evidence remains heterogeneous, limiting firm conclusions regarding causality. Nevertheless, available evidence supports that persistent activation of EBV-infected T/NK cells, endothelial injury, hyperinflammation, complement activation, and coagulation abnormalities may interact to promote vascular damage. This discussion integrates these proposed mechanisms and considers their potential contributions to CAEBV-associated vasculopathy.

### 4.1. Integrating Current Evidence

Vascular complications in CAEBV are unlikely to result from a single pathogenic mechanism. Clinical, pathological, and experimental observations suggest a multifactorial process involving persistent activation of EBV-infected T/NK cells, endothelial dysfunction, and cytokine-mediated hyperinflammation, with coagulation abnormalities and potentially complement-mediated pathways contributing in selected clinical contexts [29,38,39,40,41,44].

These processes are also unlikely to operate independently. Persistent EBV-driven immune activation and cytokine amplification may promote endothelial inflammation and vascular remodeling [29,38,39,40,41,43]. Coagulation abnormalities may further contribute to endothelial injury and thrombo-inflammatory responses [44,45,47]. Complement activation represents a possible additional pathway, although its contribution to CAEBV-associated vascular injury is supported predominantly by indirect or extrapolated evidence [49,50,51]. These mechanisms may provide a plausible framework for the heterogeneous vascular manifestations reported in CAEBV, including vasculitis, aneurysmal lesions and intracranial hemorrhage [8,10,11,21].

However, the relative contribution and temporal relationship of these pathways remain uncertain. Therefore, the proposed interactions should be regarded as a conceptual synthesis rather than an established pathogenic sequence. Studies integrating virological, immunological, vascular, and clinical data are needed to determine which mechanisms are central to CAEBV-associated vasculopathy and which represent secondary consequences of severe systemic inflammation [29,55,56].

### 4.2. Proposed Conceptual Framework

Figure 1 integrates the available evidence into three interconnected components that may contribute to vascular injury in CAEBV: CAEBV-associated vasculopathy, hyperinflammation with coagulopathy, and complement-associated microvascular injury. Persistent activation and vascular infiltration of EBV-infected T/NK cells may promote endothelial activation and vascular remodeling, consistent with pathological observations in CAEBV and experimental evidence demonstrating interactions between EBV-positive lymphoid cells and endothelial cells [8,10,40,57]. These vascular effects may be further amplified by inflammatory cytokines and other mediators released during persistent EBV-driven immune activation [29,40,58,59].

In patients with severe systemic inflammation or HLH/MAS-like hyperinflammatory states, cytokine amplification may coexist with thrombocytopenia, consumption of coagulation factors, and secondary coagulopathy, thereby creating a clinical setting in which thrombotic and hemorrhagic complications can occur [29,58]. Complement activation represents a potentially convergent pathway because complement-mediated endothelial injury can promote microvascular thrombosis and thrombotic microangiopathy, particularly in the kidney [60]. However, direct evidence establishing complement activation as a specific mechanism of CAEBV-associated thrombotic microangiopathy remains limited. Thus, its inclusion in the framework represents a biologically plausible mechanism extrapolated in part from the broader TMA literature rather than an established CAEBV-specific pathway.

Overall, Figure 1 represents a conceptual synthesis of clinical, pathological, and experimental evidence rather than a definitive causal or temporal model of CAEBV-associated vascular injury.

### 4.3. Clinical Implications

Recognition of vascular involvement in CAEBV is clinically important because vascular complications may occur in the setting of progressive systemic inflammation and multiorgan dysfunction. However, many of these manifestations are nonspecific and overlap with more common infectious, inflammatory, hematologic, and thrombotic disorders.

When otherwise unexplained systemic inflammation is persistent or progressive, EBV-associated disorders may be considered within the broader differential diagnosis. Elevated EBV DNA, hyperferritinemia, elevated soluble CD25 (sCD25), lactate dehydrogenase (LDH), and cytopenias may accompany EBV-associated HLH and other hyperinflammatory states [61,62,63,64,65]; however, these findings are not specific for CAEBV, and an elevated EBV DNA load alone cannot distinguish CAEBV from other EBV-associated conditions [3].

When CAEBV is clinically suspected, evaluation should follow contemporary diagnostic criteria. Current guidelines require not only an increased EBV DNA load but also demonstration of EBV-infected T or NK cells in peripheral blood and/or affected tissues [3]. Identification of the EBV-infected cell lineage using appropriate molecular, immunophenotypic, and EBER-based approaches may further support the distinction of systemic CAEBV from other EBV-associated disorders [3,66]. Multidisciplinary evaluation should be considered in patients with progressive multisystem involvement, particularly when vascular manifestations are present.

### 4.4. Prognosis and Long-Term Follow-Up

The clinical course of CAEBV complicated by vascular involvement appears heterogeneous, ranging from stabilization following treatment to progressive multiorgan dysfunction and death. However, because vascular complications have been reported predominantly in individual case reports and small retrospective series, their independent prognostic significance remains uncertain. Disease outcome is likely influenced by multiple factors, including the overall severity of CAEBV, systemic inflammatory activity, the extent of organ involvement, and response to treatment. Accordingly, current observations regarding prognosis should be interpreted cautiously and require validation in larger, systematically characterized cohorts.

#### 4.4.1. Predictive Biomarkers of Disease Activity and Treatment Response

Serial assessment of EBV DNA may assist in evaluating disease burden and treatment response in CAEBV [3,29,67]. In patients with concurrent hyperinflammatory features, ferritin and soluble CD25 (sCD25) may provide complementary information regarding systemic inflammation and T-cell activation, although these markers are not specific for CAEBV or vascular involvement [46,65,68]. These biomarkers should therefore be interpreted in conjunction with clinical findings, organ-specific assessments, and the overall disease course. Further studies are needed to determine whether any biomarker can specifically predict vascular complications or their outcomes.

#### 4.4.2. Prognostic Implications of Vascular Complications

Available reports suggest that extensive vascular involvement may occur in patients with severe systemic CAEBV and may accompany multiorgan complications. However, current evidence is insufficient to establish vascular involvement as an independent prognostic factor.

#### 4.4.3. Long-Term Follow-Up

Long-term surveillance is appropriate for patients with CAEBV because persistent or recurrent disease activity and progression of EBV-positive T/NK-cell lymphoproliferative disease may occur. Serial assessment of EBV-DNA and selected inflammatory markers, including ferritin and sCD25 when clinically indicated, may assist in monitoring disease activity. In patients with previous vascular or organ involvement, follow-up should additionally be tailored to residual or recurrent neurological, renal, cardiovascular, and hematologic abnormalities. Because standardized surveillance protocols specifically for CAEBV-associated vascular complications have not been established, the frequency and extent of follow-up should be individualized according to disease activity, treatment status, and the organs involved.

### 4.5. Knowledge Gaps and Future Directions

Despite increasing recognition of vascular manifestations in CAEBV, substantial uncertainties remain regarding their frequency, pathogenesis, natural history, and prognostic significance. Most available evidence is derived from individual case reports and small retrospective series, and many reports predate or do not fully document contemporary diagnostic criteria for CAEBV. These limitations make it difficult to distinguish vascular manifestations directly attributable to CAEBV from those associated with overlapping hyperinflammatory, thrombotic, or other EBV-related conditions.

#### 4.5.1. Key Knowledge Gaps

One major unresolved question is how EBV-infected T/NK-cell disease contributes to vascular injury. Although endothelial dysfunction, cytokine-mediated inflammation, coagulation abnormalities, and complement activation have been proposed as potential mechanisms, their relative contributions remain incompletely established.

#### 4.5.2. Future Research Directions

Future studies should prioritize multicenter registries and prospective cohorts using contemporary diagnostic criteria for CAEBV to define the incidence, clinical spectrum, and prognostic significance of vascular complications. Longitudinal assessment of EBV-DNA together with markers of inflammation, endothelial injury, coagulation, and complement activation may improve risk stratification, while EBER in situ hybridization and emerging spatial or single-cell approaches may help clarify the underlying vascular mechanisms. Further translational and clinical studies are also needed to determine whether targeted immunomodulatory approaches have therapeutic value in CAEBV-associated vascular complications.

## 5. Conclusions

Vascular complications are uncommon but potentially severe manifestations of chronic active Epstein–Barr virus (CAEBV) disease and may involve multiple organ systems. Available evidence suggests that vascular injury may arise from interactions among EBV-infected T/NK-cell-driven immune dysregulation, hyperinflammation, endothelial dysfunction, and thrombo-inflammatory pathways. However, the relative contributions of these mechanisms remain incompletely defined.

This review summarizes the clinical spectrum and current mechanistic understanding of vascular complications in CAEBV and highlights important diagnostic and prognostic uncertainties. The proposed conceptual framework illustrates how interconnected inflammatory and vascular pathways may contribute to multisystem vascular injury rather than establishing a single causal mechanism. Further mechanistic studies and well-characterized multicenter cohorts using contemporary diagnostic criteria for CAEBV are needed to clarify these relationships, identify clinically useful biomarkers, and improve the diagnosis and management of affected patients.

## Figures and Tables

**Table 1:** Representative vascular manifestations reported in CAEBV and their proposed pathogenic mechanisms.

Organ System	Representative Vascular Manifestations	Proposed Pathogenic Mechanisms
Cardiovascular/systemic arteries [7,10,18]	Coronary artery dilatation/aneurysm; aortic and systemic arterial dilatation/aneurysm; pulmonary arterial hypertension	Vasculitis, vasculopathy, vascular wall and endothelial injury
CNS [8,11,19,20,21]	Cerebral vasculitis/vasculopathy; cerebral artery aneurysm; intracranial hemorrhage secondary to aneurysmal rupture	Vascular inflammation and vascular wall injury
Cutaneous vessels [8,14,15]	Cutaneous/systemic vasculitis and vasculitic skin lesions	EBV-infected T/NK-cell infiltration and vascular inflammation

CNS; central nervous system, EBV; Epstein–Barr virus.

## Data Availability

No new datasets were generated or analyzed in this study. Data sharing is not applicable to this article.
